# Marked Neutrophilic Cerebrospinal Fluid Pleocytosis Following Intravenous Immunoglobulin Mimicking Bacterial Meningitis

**DOI:** 10.7759/cureus.111792

**Published:** 2026-06-30

**Authors:** Zaw Lwin Show, Jeevan Perera, Thet Wint Htae, Indiketi H Jayasiri

**Affiliations:** 1 Acute Medicine, North Cumbria Integrated Care NHS Foundation Trust, Carlisle, GBR

**Keywords:** acute inflammatory demyelinating polyneuropathy (aidp), aseptic meningitis, bacterial meningitis mimic, campylobacter infection, cerebrospinal fluid, cerebrospinal fluid analysis, drug-induced aseptic meningitis, guillain-barré syndrome (gbs), intravenous immunoglobulin (ivig)

## Abstract

Intravenous immunoglobulin (IVIG) is an established therapy for immune-mediated neurological disorders and is generally well-tolerated, although rare neurological adverse effects have been reported. We describe a woman in her 40s treated with IVIG for suspected acute inflammatory demyelinating polyneuropathy following *Campylobacter* infection.

Within 48 hours of therapy, she developed a headache and fever without meningism. Neuroimaging was unremarkable. Cerebrospinal fluid (CSF) analysis demonstrated marked pleocytosis with neutrophilic predominance, initially suggestive of bacterial meningitis. However, inflammatory markers and microbiological investigations were negative. Symptoms resolved rapidly following the discontinuation of IVIG without antimicrobial therapy. A diagnosis of IVIG-induced aseptic meningitis was made.

This case highlights that IVIG can produce marked CSF pleocytosis mimicking bacterial meningitis and underscores the importance of clinical correlation to avoid unnecessary antimicrobial treatment.

## Introduction

Intravenous immunoglobulin (IVIG) is widely used in the treatment of immune-mediated neurological disorders, including Guillain-Barré syndrome and acute inflammatory demyelinating polyneuropathy, and is generally considered safe and well-tolerated [1,2]. Although most adverse effects are mild and self-limiting, including headache, fever, chills, myalgia, and infusion-related reactions, uncommon but clinically significant neurological complications such as aseptic meningitis have been reported [1-4].

IVIG-associated aseptic meningitis is an uncommon adverse event, with a reported incidence of approximately 0.6-1% among treated patients [3]. It typically develops within 24-48 hours of IVIG administration and may closely resemble bacterial meningitis, presenting with headache, fever, photophobia, neck stiffness, and cerebrospinal fluid (CSF) pleocytosis [1,3-5]. Because of this overlap, patients are frequently investigated for central nervous system infection and may receive unnecessary antimicrobial therapy before the diagnosis is recognised [1,4,5].

Previous reports have demonstrated variable CSF findings, ranging from lymphocytic to neutrophilic pleocytosis, making differentiation from bacterial meningitis particularly challenging [1,5,6]. Recognition of the temporal relationship between IVIG administration, sterile CSF microbiology, and rapid clinical resolution is therefore essential to establish the diagnosis and avoid unnecessary treatment [1,5,7].

We report a patient with suspected acute inflammatory demyelinating polyneuropathy who developed IVIG-associated aseptic meningitis characterised by marked neutrophilic CSF pleocytosis, highlighting an important diagnostic pitfall for clinicians managing patients receiving IVIG.

## Case presentation

A woman in her 40s presented with a one-week history of progressive symmetrical weakness affecting all four limbs. These symptoms followed a recent gastrointestinal illness, during which stool testing confirmed *Campylobacter *species infection.

On examination, she had symmetrical weakness in all four limbs (Medical Research Council grade 3/5) with preserved cranial nerve function. Although she reported tingling sensations, there were no objective sensory signs on examination. There were no signs of respiratory compromise.

Following neurology review, a working diagnosis of acute inflammatory demyelinating polyneuropathy was made, and empirical treatment with IVIG was initiated.

Approximately 48 hours after the commencement of treatment, the patient developed a headache and fever lasting 2-3 hours. There were no features of meningism, photophobia, or altered consciousness. She remained haemodynamically stable.

Given the temporal relationship with IVIG administration, infection was initially considered; however, the absence of meningism and the transient nature of symptoms raised suspicion for a non-infective cause.

Inflammatory markers, including C-reactive protein, were within normal limits.

Neuroimaging (computed tomography (CT) venography and magnetic resonance imaging (MRI) of the brain and whole spine) demonstrated no acute abnormalities (Figure 1).

**Figure 1:**
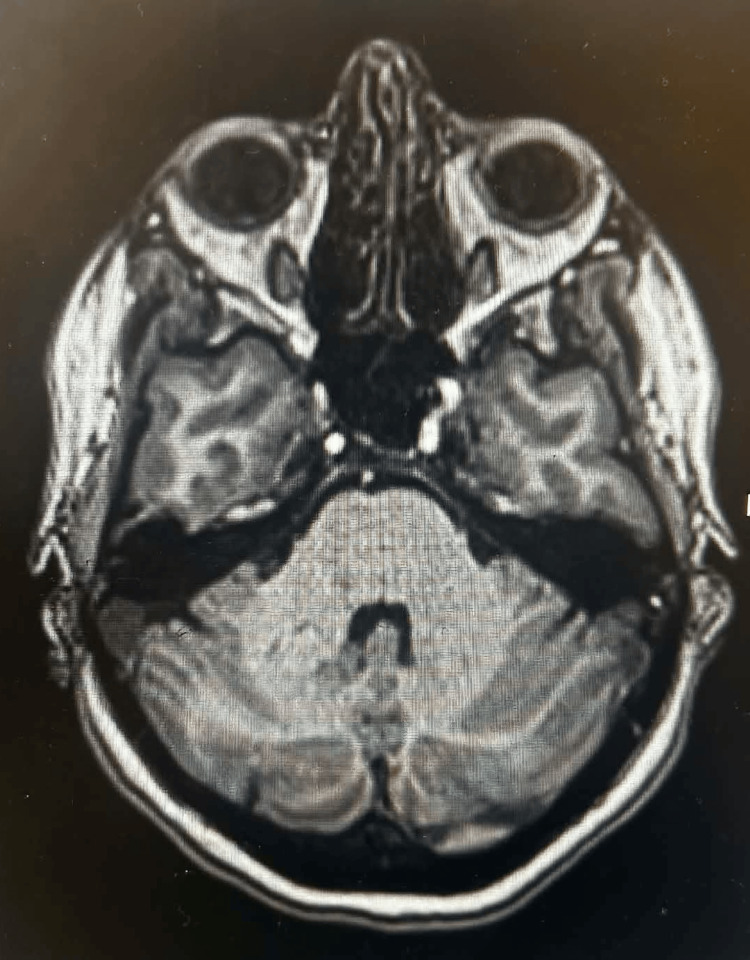
Axial T1-weighted magnetic resonance imaging of the brain demonstrating no acute intracranial abnormality

Lumbar puncture revealed clear CSF with a white cell count of 1310 cells/mm³, comprising 60% polymorphs and 40% lymphocytes. Red blood cells were absent (0 cells/mm³). No organisms were seen on microscopy, and bacterial cultures demonstrated no growth. Viral polymerase chain reaction testing was negative.

Key investigation findings are summarised in Table 1.

**Table 1:** Summary of investigations Summary of key investigations performed during the assessment of suspected intravenous immunoglobulin-associated aseptic meningitis. CSF analysis demonstrated marked pleocytosis with neutrophilic predominance in the absence of microbiological evidence of infection. CSF: cerebrospinal fluid; CT: computed tomography; MRI: magnetic resonance imaging

Investigation	Result
C-reactive protein	Within normal limits
CT venography	No acute abnormality
MRI of the brain	No acute abnormality
MRI of the whole spine	No acute abnormality
CSF white cell count	1310 cells/mm³
CSF differential	60% polymorphs, 40% lymphocytes
CSF red blood cells	0 cells/mm³
Bacterial culture	No organisms seen; no growth
Viral polymerase chain reaction	Negative

The markedly elevated CSF white cell count with neutrophilic predominance initially raised concern for bacterial meningitis. However, the absence of systemic inflammatory response, sterile CSF, and rapid spontaneous resolution made infection unlikely.

IVIG was discontinued after discussion with the patient and the neurologist. No antimicrobial therapy was initiated, given the low clinical suspicion for bacterial infection. Supportive therapy including physiotherapy was provided.

The patient's headache and fever resolved spontaneously within a few hours without specific treatment. She remained clinically stable with no recurrence of symptoms. Her neurological symptoms demonstrated gradual improvement, and she was discharged following a period of observation.

## Discussion

Aseptic meningitis is a recognised adverse effect of IVIG therapy, typically occurring within 24-48 hours of administration [1-4]. The reported incidence is approximately 0.6-1% among treated patients [3]. A recent narrative review highlighted the rarity of this complication despite widespread IVIG use [1].

The mechanism remains incompletely understood but may involve hypersensitivity reactions, cytokine-mediated inflammatory responses, or direct meningeal irritation [1-4].

CSF findings in IVIG-associated aseptic meningitis are variable and may demonstrate either lymphocytic or neutrophilic predominance [1,5-8]. In some cases, CSF pleocytosis may be marked and closely mimic bacterial meningitis [3-5]. White cell counts exceeding 1000 cells/mm³ have been reported but remain relatively uncommon [1].

IVIG-associated aseptic meningitis has been reported in patients treated for Guillain-Barré syndrome, acute inflammatory demyelinating polyneuropathy, Kawasaki disease, dermatomyositis, renal transplantation, and other immune-mediated conditions [5,6,8-11]. Recognition is important to avoid misdiagnosis, unnecessary antimicrobial therapy, prolonged hospital admission, and additional invasive investigations [1].

Differential diagnoses considered included bacterial meningitis, viral meningitis, cerebral venous sinus thrombosis, subarachnoid haemorrhage, and progression of acute inflammatory demyelinating polyneuropathy. Bacterial meningitis was considered because of the marked neutrophilic CSF pleocytosis. However, the absence of meningism, normal inflammatory markers, sterile CSF microscopy and culture, and rapid spontaneous clinical resolution without antimicrobial therapy made bacterial infection unlikely. Viral meningitis was considered less likely because viral polymerase chain reaction testing was negative and the clinical course was self-limiting. Cerebral venous sinus thrombosis and other structural intracranial pathology were excluded by CT venography and MRI of the brain, which demonstrated no acute abnormalities. Progression of acute inflammatory demyelinating polyneuropathy did not explain the acute onset of headache, fever, and inflammatory CSF findings following IVIG administration.

The close temporal relationship between IVIG administration and symptom onset, together with sterile microbiological investigations, normal inflammatory markers, and rapid spontaneous resolution, strongly supported the diagnosis of IVIG-induced aseptic meningitis. These findings are consistent with previously reported cases in patients receiving IVIG for acute inflammatory demyelinating polyneuropathy and other immune-mediated disorders [1,5,8-11].

## Conclusions

IVIG is an effective therapy for immune-mediated neurological disorders but can rarely cause aseptic meningitis with marked CSF pleocytosis that closely mimics bacterial meningitis. Recognition of this complication is essential to avoid unnecessary antimicrobial therapy and invasive investigations. Careful clinical assessment, timing of symptom onset, and microbiological results remain crucial in guiding diagnosis and management.
